# Adolescent-Parent Communication on Sexual and Reproductive Health issues in Ethiopia: A Systematic Review and Meta-analysis

**DOI:** 10.4314/ejhs.v30i5.22

**Published:** 2020-09

**Authors:** Akine Eshete, Sisay Shewasinad

**Affiliations:** 1 College of Health Sciences, Department of Public Health, Debre Berhan University, Ethiopia; 2 College of Health Sciences, Department of Nursing, Debre Berhan University, Ethiopia

**Keywords:** Adolescent, Communications, Sexual and reproductive health issues, determinant factors, Ethiopia

## Abstract

Adolescent-parent sexual communication is an effective strategy to make healthy decisions, delay and protect from risky behavior. Thus, this systematic review and meta-analysis aimed to synthesize and estimate the level of adolescent- parent communication on sexual and reproductive health issues in Ethiopia.

**Methods:**

Cross-sectional studies were systematically searched using databases such as PubMed, Google Scholar, Cochrane Library and gray literature. Information was extracted using a standardized form of JBI. Data were analyzed using the ‘meta’ packages of the Stata software (version 11.0). I-squared statistic was applied to check the heterogeneity of studies. Funnel plot and Egger's test were used to check for publication bias. P-value <0.05 on the Egger test was considered indicative of statistically significant publication bias.

**Results:**

A total of 359 articles were identified, of which 19 were eligible for meta-analysis. Adolescent-parent communications on SRH issues were significantly reported within the range of 25.3% to 36.9% and more preferred to discuss with their friends. The overall pooled level of adolescent parent communication was 40.70 (95%CI: 34.26–47.15). Adolescents who lived in urban areas, having good knowledge of SRH issues, adolescents who agreed on the importance of discussion and adolescents who ever had sexual intercourse were more likely to discuss SRH issues with their parents.

**Conclusion:**

The overall pooled level of adolescent-parent communication was 40.70%, and also adolescent-parent communications were dominantly reported with the ranges of 25.3% to 36.9%. Being urban dweller, being knowledgeable, and being agreed on the importance of discussion were significantly associated with adolescent-parent communication. Cultural taboo, shame and lack of communication skills were reasons that hindered communication between parents and adolescents. Therefore, program implementers should work to increase adolescent-parent communication.

## Introduction

Adolescents and young adults have been overlooked in global health, due to the fact that they have had fewer health gains than other age groups. While adolescents' health issues are preventable, they face multiple barriers in accessing sexual and reproductive health (SRH) issues information (1). Reproductive health information is not uniformly available for all adolescents worldwide, particularly in developing countries. The extent to which adolescents are knowledgeable about SRH issues is determining to make decisions about SRH issues (2).

During adolescence and young age, independence and autonomy from family increases and peer relations become more significant. At this time, the family has a great impact on adolescent health and protects them from multiple health risks. Families can act as a powerful gatekeeper for access to education, health services, and other resources (3–5).

Sexual health education and open positive parent-adolescent communication on SRH issues in homes are important strategies to prevent reproductive health problems. A systematic review in Africa shows adolescents who had no communication on SRH issues with their parents were more likely to start childbearing (6). Early childbearing and unintended pregnancies were highly associated with parenting attitudes, behavior and styles (7). As evidence suggests, adolescent-parent communication is an effective strategy among several strategies that improve healthy sexual and reproductive health behavior (8). Adolescents who discuss with their parents on SHR issues are more likely to make healthy decisions on the use of reproductive health services, delay sexual activity, protect from risky behavior and support the healthy sexual socialization of adolescents (9).

In a systematic review in Ethiopia, adolescents who ever discussed with family, relatives, and healthcare workers were more likely to utilize SRH services than adolescents who never discussed SRH issues with anyone. Besides, discussion on SRH issues potentially increases knowledge and awareness among adolescents and motivates them to utilize the available SRH services (10).

Parent-adolescent sexual communication has received attention as a protective factor against a delay in sexual activity, protect from risky sexual behavior and maintain a healthy behavior of adolescents (8). However, until now, the body of evidence on sexual communication among adolescents in Ethiopia had yet to be synthesized. There have been several epidemiological studies available on adolescent-parent communication on SRH issues in Ethiopia. Studies revealed a wide level of variation on adolescent-parent communication on SRH issues over time and across geographical areas (11–31). These wide variations across geographical areas in Ethiopia may not produce credible evidence for planners, policymakers, implementers and service providers. Therefore, this systematic review and meta-analysis was aimed to synthesis, and to estimate the level of adolescent-parent communication on sexual and reproductive health issues and its determinant factors. Ultimately, the findings of this systematic review and metaanalysis will be used to update planners, policymakers and service providers in a way that increases adolescent-parent communication strategies.

The objective of this systemic review and meta-analysis is to determine the level of adolescent-parent communication on sexual and reproductive health issues and its determinant factors. Accordingly, the review intends to answer the research question: “What is the best available evidence on the level of adolescent-parent communication on sexual and reproductive health issues and determinant factors of adolescent parent communication in Ethiopia?”

## Methods

**Study settings:**: This systematic and meta-analysis was conducted in Ethiopia. Ethiopia has nine regions; namely, Tigray, Afar, Amhara, Oromia, Somali, Benishangul-Gumuz, Southern Nations Nationalities and People Region (SNNPR), Gambella, Harari, and two Administrative states (Addis Ababa City Administration and Dire-Dawa City Administration). Studies were included in the review according to the following criteria.

## Inclusion Criteria

**Type of studies**: All published and unpublished cross-sectional studies including government reports related to adolescent-parent communication on SRH issues were included. Articles were included if they fulfilled the following criteria, study type: full-text cross-sectional articles written in English which have been published between 2000 and 2019 in peer reviewed journals, primary journals, be on human subjects.

**Study participants**: In- and out-of-school adolescents aged between 10 and24 years.

**Types of outcome measures (communication on SRH issues)**: Adolescents who discussed at least two SRH issues with their parents.

**Exclusion criteria**: Citations without abstracts and/or full texts, commentaries, anonymous reports, letters, duplicate studies.

**Search strategy and information sources**: The database search had been structured using CoCoPop, where, Context (Ethiopia), condition (level of adolescent-parent communicating on SRH issues), Population (adolescent age group 10–24 years). The search strategies were developed using Boolean operators. Notably, to fit the advanced PubMed database, the following search strategy applied; (Level OR “Level of Adolescent-Parent Communication” OR “Level of Adolescent-Parent Communication on Sexual and Reproductive Health issues” OR “Adolescent Communication on Sexual and Reproductive Health issues” OR “Adolescent Communication on Sexual and Reproductive Health issues with their parent” OR “Parent communication on sexual and reproductive Health issues”) AND (Adolescent OR Adolescents OR “Adolescents age 10–19 years” OR “Adolescents age 10–24 years”) AND (Determinant OR Determinants OR “Determinant factor” OR “Determinant factors” OR Factor OR Factors OR “Associated factor” OR “Associated factors”) AND (Ethiopia OR “Northern Ethiopia” OR “Southern Ethiopia” OR “South east Ethiopia” OR “South west Ethiopia” OR “Amhara region” OR “Oromiay region” OR SNNPR OR “Tigray region” OR “DiraDawa” Harar OR “Gambella region” OR “Benishangul-Gumuz Region” OR “Addis Ababa”) AND (“2000/01/01”[PDat] : “2019/12/31”[PDat] ) AND Humans[Mesh].

The presence of precursor systematic review and/or protocol on the topic of interest was checked on the Cochrane database of a systematic review and Joanna Briggs Institute database of a systematic review. However, PROSPERO registration was not done.

Electronic database searches were conducted using PubMed, Google Scholar and Cochrane library and research gate from October 2019 to November 2019. The search focused on all published and unpublished studies with the cross-sectional study on adolescent-parent communication on sexual and reproductive health issues in Ethiopia. The authors were contacted and requested for full articles by email when the article was not accessed from these sources. To minimize time-lag bias, the search process was updated on November 18, 2019.

**Quality assessment tool**: Retrieved studies were exported to endnote version 7 to remove duplicate studies. A search strategy was done by the corresponding author. Both authors were blinded to the journal, authors, and results. There were no conflicts in final selection decisions. The selections of identified studies were done in two stages. In the first stage, a selection of relevant studies was selected based on titles and abstracts. In the second stage, studies that met the inclusion criteria and the full paper found for detailed assessment based on the inclusion criteria were considered.

Two reviewers (SS and AE) performed the study eligibility assessment independently by using JBI checklists (The Joanna Briggs Institute, Joanna Briggs Institute Reviewers' Manual)**.** A critical appraisal checklist for cross-sectional studies was adopted by JBI and used to assess the overall methodological quality and evaluated the risk of bias (Table 1).

**Table 1:** Description of the included studies

Authors,	Study area (regions)	Sample	Adolescent parent communication on SRH issues, n (%)	JBI-Quality score
Tesso et al., 2012	Oromia	2269	737(32.5%) had discussion with their parents	88.9
Shiferaw K et al., 2014	Amhara	688	254(36.9%) had discussion with their parents	100
Ayalew et al., 2014	Dire Dawa	641	236(36.8%) had discussion with their parents	100
Ayehu et al., 2016	Amhara	781	189(25.3%) had discussion with their parents	100
Dessie Y. et al, 2015	Harar	4559	1409 (30.91%) had discussion with their parents	88.9
Cherie N. et al., 2018	Amhara	332	267(82.7%) had discussion with their parents	88.9
Yadeta et al. 2014	Harar	751	216(28.76%) had discussion with their parents	88.9
Yesus DG. et al, 2010	Benishangul Gumuz Region	412	119(28.9%) had discussion with their parents	88.8
Taddele M. et al., 2018	Amhara	394	114(28.9%) had discussion with their parents	88.9
Mekie M. et al., 2019	Amhara	394	270(68.5%) had discussion with their parents	88.9
Shewasinad S. et al., 2017	SNNP	356	103(28.9%) had discussion with their parents	77.8
Melaku YA. et al., 2014	Tigray	807	351(43.5%) had discussion with their parents	100
Habte NM. et al., 2019	oromia	394	186(47.2%) had discussion with their parents	88.9
Kusheta et al., 2019	SNNP	411	144(35%) had discussion with their parents	88.9
Yohannes Z. et al., 2015	SNNP	660	390(59.1%) had discussion with their parents	88.9
Mekonen MT, et al, 2018	Amhara	674	205(30.4%) had discussion with their parents	88.9
Zemenu Y. et al., 2016	Tigray	521	300(57.6%) had discussion with their parents	88.9
Nurilign A. et al., 2013	Tigray	488	155(31.8%) had discussion with their parents	88.9
Martha F., 2009	SNNP	694	205(30.4%) had discussion with their parents	88.9
Fanta M, et.al., 2016	SNNP	740	301(40.7) had discussion with their parents	88.9

**Figure 1:**
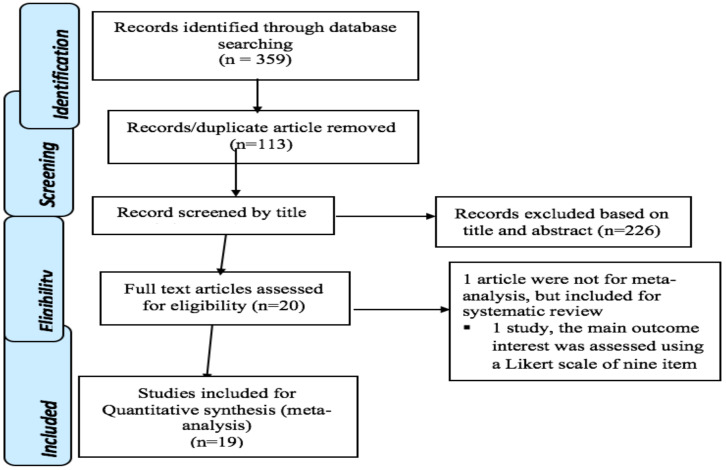
the PRISMA flow diagram of identification and selection of studies for the systematic review and meta-analysis

**Data extraction**: A standardized data extraction form of JBI was used to extract the necessary data The JBI data extraction form contains; sample frame, study participants, sample size, study subjects and setting, type of data analysis, measurement of tools. The data extraction tool was piloted by considering the inclusion criteria to check consistency and to ensure that all the relevant information was captured. The extraction tool includes the title of the study, the first author's name, and the year of publication (Table 1). During the extraction process, data discrepancy among data extractors was resolved by referring back to the original study. The screening and selection process of the reviewed articles was summarized using the PRISMA flow chart (Figure 1 (32).

**Data synthesis and statistical analysis**: Data were analyzed using the ‘meta’ packages of the Stata software (version 11.0). The quantitative data synthesis method was used to present the data extracted data from each study. Heterogeneity among the studies was evaluated using the χ2 test on Cochrane's Q statistic (32), and I-square estimate greater than >75% was considered as indicative of moderate to high levels of heterogeneity (33). Subgroup analysis was done to explore differences in outcomes according to a study area (region). The funnel plot and Egger's test were used to check the presence of publication bias (34). P-value <0.05 on the Egger test was considered indicative of statistically significant publication bias.

## Results

**Description of the included studies**: The search strategy retrieved 359 studies from different databases. About 113 articles were excluded because of duplication. After removing duplicates, a total of 246 studies were retrieved of which 225 were rejected by reading their titles and abstracts. Finally, 20 studies were screened for full-text and included for the systematic review. Only nineteen studies were included for meta-analysis. All studies that reported the level and determinants of adolescent-parent communication on sexual and reproductive health issues were included for the final review (Figure 1).

**Characteristics of included studies**: Full-text cross-sectional articles written in English and published from 2000–2019 were studied in a different part of Ethiopia; of twenty studies, 6 (30%) of them were done in Amhara region (12, 14, 16, 19, 20, 28), 5 (25%) in Southern Nation Nationality People Region (SNNPR) (21, 24, 26, 30, 31), 3(15%), 3(15%) in Oromia region (11, 23, 27), 3(15%) in Tigray (22, 25, 29), 2(10%) in Harar region (15, 17), 1(5%) in Benishangul Gumuz region (18) and 1(5%) in Dire Dawa (13). In the included articles, 14 articles were school-based cross-sectional studies whereas 6 of them were community-based cross-sectional studies. Among 20 included studies, only one study was unpublished (30).

In the included studies, the mean age of adolescents ranged from 17.1(±1.5 SD) to 19.33 (± 1.72 SD) years. The sample size of the included studies ranged from 332 (16) and 2269 (11). A total of 17,611 adolescents were included in all studies. The summary of all relevant features and main findings of the including studies are presented in (Table 1).

**Parents-adolescents communication on sexual and reproductive health issues**: In most reviewed studies, the level of adolescent-parent communication on SRH issues was dominantly reported within the ranges from 25.3% to 36.9% (11–15, 17–19, 21, 24, 28–30). In one study, the level of communication on the SRH issue was high which accounted for 82.7% (16).

In the review, it was found that most of the female adolescents had communication on SRH issues with their parents, reported with the ranges from 52.1% to 65.1% (13, 14, 16, 21, 23–25).

**Adolescent-parents communication on different sexual and reproductive health issues communication on STI/HIV/AIDS**: Most of the adolescents held communication on STI/HIV/AIDS with their parents, reported with the range of 51.8% to 96.8% (12–14, 16–18, 21, 22, 24, 26, 28, 30, 31).

**Communication on unwanted pregnancy**: In the included studies, the majority of the studies reported that less than one-third of adolescents had communication on associated risk aspect of unwanted pregnancy (12–14, 17, 19, 22, 26, 28, 30, 31). In three studies, more than half (52.9% to 56.2%) of adolescents had communication with their parents on unwanted pregnancy (16, 18, 21, 31).

**Communication on condom and contraceptive methods**: Around 7 studies reported that lower proportion of adolescents had communication with their parents on pregnancy prevention methods such as condom and contraception method that ranges from 14.8% to 36.2% (12–14, 19, 26, 28, 30). Specifically, for the contraception method, in six studies, more than half of the adolescents (51% to 79.9%) had communication with their parents (13, 16, 17, 21, 24, 31).

**Communication on body change during puberty**: In most of the individual studies, most of the adolescents who ever discussed on puberty with their parents reported with the range of 11.6% to 39.7% (11, 17, 22, 26, 30). In two studies, more than two-thirds of adolescents had discussions with their parents on puberty (75.0% and 78.6%)(16, 18).

**Communication on avoiding premarital sex**: In the included studies, in five studies, half of the adolescents had a discussion with their parents on avoiding premarital sex, with the range of 52% to 57% (13, 16, 18, 21, 31). In three studies, adolescents who ever discussed avoiding premarital sex with their parents reported with the range of 15.9 % to 32.0% (12, 14, 28, 30, 35).

**Gender preference for adolescent-parent communication**: In the included studies, most adolescents preferred communication with mothers (11, 13, 14, 16, 18, 20, 22, 23). And, the majority of adolescents preferred to communicate with their friends (11, 14, 16, 18, 23) and sisters (11, 12, 14, 16, 22, 23, 26).

**Factors associated with adolescent-parent communications**: In three reviewed articles (14, 24, 29), males were more likely to discuss on different SRH issues with their parents. In four individual studies, adolescents who lived in urban areas were more likely to discuss different SRH issues with their parents (12, 14, 16, 29). Adolescents who agreed on the importance of discussion on SRH issues (12, 20, 28, 31), whoever got SRH information (13, 24, 28), having good knowledge on SRH issue (17, 19, 24, 29, 31) and who ever had sexual intercourse (12, 28) were more likely to discuss on SRH issues with their parents.

## Meta-Analysis Results

The drive of this meta-analysis was to estimate the pooled level of adolescent-parent communication on sexual and reproductive health issues in Ethiopia, by using proportions. A total of 19 studies met the inclusion criteria for meta-analysis.

**Figure 2:**
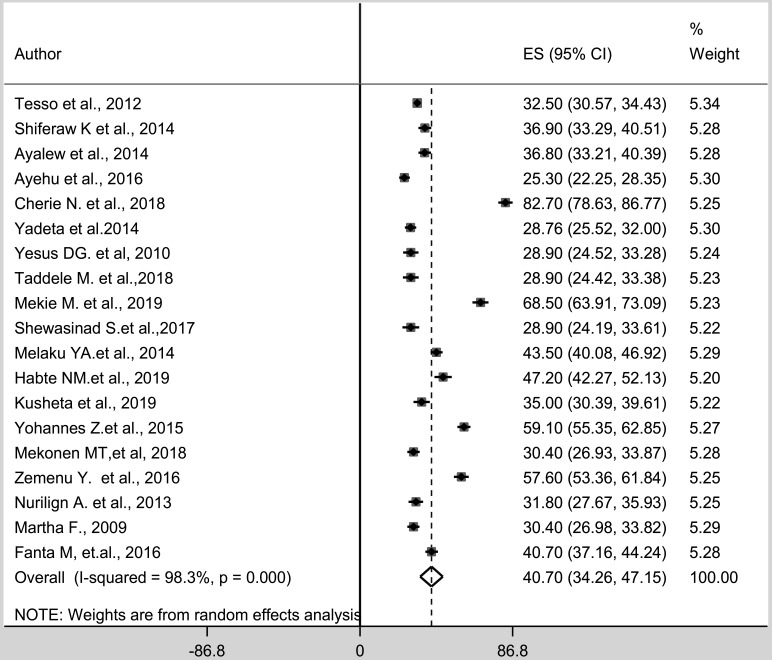
*Pooled level of* adolescent-parent communication on sexual and reproductive health issues *in Ethiopia from 2000–2019*

The estimated overall level of adolescent-parent communication on SRH issues using the random effect model was 40.70 (95%CI: 34.26–47.15). The weights of studies using the random-effect model ranged from 4.93 to a maximum of 5.08. The presence of heterogeneity among the studies was tested using I-squared statistics. I-squared (I^2^) statistics was (I^2^ = 98.3) (p=<0.0001) (Figure 2).

**Factors associated with adolescent parent communications**: Adolescents who lived in urban areas (OR = 2.14, 95% CI: 1.39–3.39), having good knowledge on sexual and reproductive health issue (OR = 1.99, 95% CI: 1.02–3.88), adolescents who agreed on the importance of discussion on SRH issues (OR = 3.57, 95% CI: 2.17–5.88) and adolescents who ever had sexual intercourse (OR = 1.70; 95% CI 1.25–2.30) were more likely to discuss on different SRH issues with their parents (Table 2).

**Table 2:** Factors associated with adolescent parent communications on sexual and reproductive health issues in Ethiopia from 2000 to 2019

Variables	OR (95% CI)	Heterogeneity
Being a male adolescent	1.12 (0.74–1.69)	I^2^=75.1, p=0.0001
Adolescents who reside in urban areas	**2.14 (1.39–3.39)**	I^2^=67.8, p=0.014
Adolescents who agreed on the importance of discussion on SRH issues	**3.57 (2.17–5.88)**	I^2^=63.5, p=0.027
Adolescents whoever got SRH information	2.04 (0.65–6.54)	I^2^=0.0, p=0.998
Having good knowledge of a sexual and reproductive health issue	**1.99 (1.02–3.88)**	I^2^=0.0, p=0.685
Ever had sexual intercourse	**1.70 (1.25–3.30)**	I^2^=0.0, p=1.00

**Evaluation for publication bias**: The presence of heterogeneity among the studies was tested using I-squared statistics. I-squared (I^2^) statistics for level of adolescent-parent communication on SRH issues was ((I2 = 98.3) (p=<0.0001)). I2 = 98.3 and (p=<0.0001) indicates the presence of significant heterogeneity among the included studies (Figure 1).

**Figure 3:**
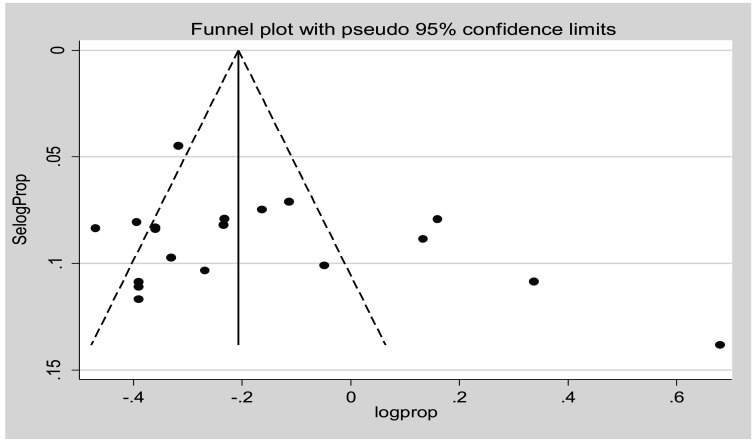
Funnel plot of effect estimates against standard error of log estimate

**Figure 4:**
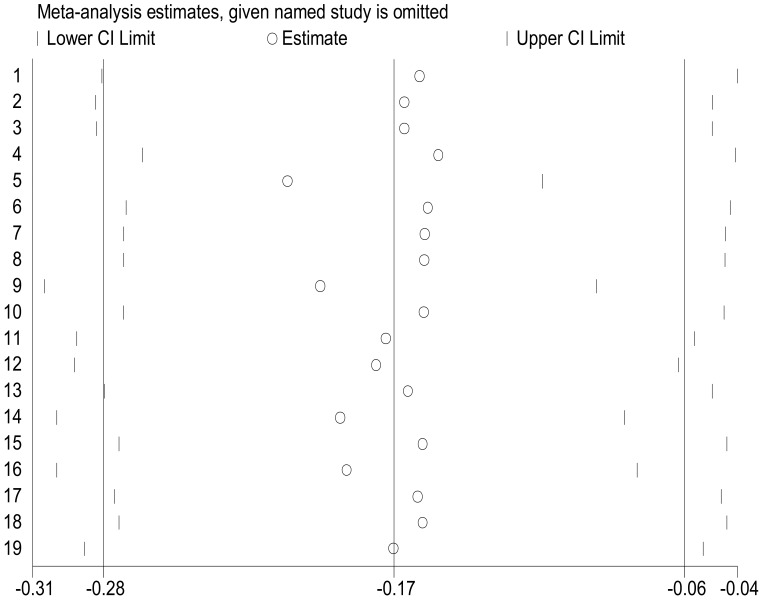
Plot of sensitivity analysis to assess the influence of individual study

The funnel plot was unsymmetrical and the distribution of studies indicates the presence of publication bias. More studies are found on both sides of the funnel plot margin (Figure 3). Egger's test was performed and the estimated bias coefficient (intercept) is 3.399 with a standard error of 2.46, giving a p-value of 0.185. The test thus proves that there is no statistical evidence of publication bias. Sensitivity analysis was performed to identify the influence of individual studies on the estimates. The test indicated that there was no single study that affected the estimates (Figure 4).

## Discussion

This systematic review and meta-analysis was aimed to synthesis and pool the level of adolescent-parent communication on sexual and reproductive health issues and its determinant factors. A total of twenty-one studies fulfilled the inclusion criteria for a systematic review.

This current review found a difference in the level of adolescent-parent communication by geographical and gender differences. The review indicates that adolescent-parent communication on SRH issues was dominantly reported with the ranges of 25.3% to 36.9% (11–15, 17–19, 21, 24, 28–30), and female adolescents had better communication with their parents (13, 14, 16, 21, 23, 24). In a meta-analysis, the estimated overall level of adolescent-parent communication on SRH issues using the random effect model was 40.70 (95%CI: 34.26–47.15). Understanding the barriers of adolescent-parent communication was critical to formulating effective policies and programs. Lessons from different studies in Ethiopia revealed that sexual communication is considered as cultural taboos topics, being ashamed, lack of communication skill of adolescent, and parents lack communication skill that makes them not to discuss openly with their parents (11–31), which was consistent with a systematic review in African countries (9). Hence, parental knowledge on sexual issues, initiation of family life education and access to SRH information for both adolescents and parents are needed.

This systematic review showed that adolescents mostly held discussions on STI and HIV/AIDS with their parents compared to other topics of SRH issues (12–14, 16–18, 21, 22, 24, 26, 28, 30, 31); this finding was supported by evidence found in developing countries (2).

Therefore, the review result indicates that adolescents had lack comprehensive knowledge on SRH issues across its different dimensions of SRH issues. Taking a unified approach to SRH issues would help to equalize knowledge across various dimensions of SRH and there is a need to equip and educate parents and adolescents on different SRH issues.

In this review, adolescents were more comfortable to discuss with their friends, yet of their parents preferred their mothers (11, 12, 14, 16, 18, 20, 21, 23, 26). This indicates that adolescents preferred to receive information from their peer friends compared to their parents. This could imply that if peers are not equipped with the right information, they may influence their friends negatively. There is a need to equip friends on sexual issues to avoid such a negative impact on adolescents' sexual behavior. In reality, parents are a powerful influence in the lives of their children; they influence their children's decisions about SRH issues.

Evidence suggested that open parent-adolescent communication in a home is an effective strategy among several strategies that improve healthy sexual and reproductive health behavior (8). To make healthy decisions on the use of reproductive health services and delay sexual activity, protect from risky behavior and support the healthy sexual socialization of adolescents (9).

Generally, the estimated overall pooled level of adolescent-parent communication on SRH issues in Ethiopia was 40.70 (95%CI: 34.26–47.15). Adolescents who lived in urban areas, having good knowledge of SRH issues, adolescents who agreed on the importance of discussion on SRH issues and adolescents who ever had sexual intercourse were more likely to discuss SRH issues with their parents.

Adolescent-parent communication on SRH issues was dominantly reported with the ranges of 25.3% to 36.9%. The findings of this systematic and meta-analysis finding indicates a significant proportion of adolescents did not discuss with their parents. Several barriers to adolescent-parent communication were identified, which should be taken into consideration in the development of interventions and programs. Cultural taboo, shame and lack of communication skills were reasons that hindered communication between parents and adolescents. Therefore, for effective parent-adolescent communication on SRH issues, there is a need to address the barriers that hinders communication.

Adolescent-parent communication has been an effective strategy against adolescent sexual risk-taking behavior. Yet, in this review, adolescents preferred their peer friend than parents to discuss different SRH issues such as STI and HIV/AIDS. Adolescent comprehensive knowledge of different components of SRH issues and information on different sexual and reproductive health is vital to support adolescents' decision-making process.

In the light of these challenges, the country needs to strengthen the implementation of the Health Extension Program and strengthen the health development army in the community. The government needs to work with the private sector and nongovernmental providers that will improve parent-adolescent communication on SRH issues in the country. Strategies are needed to implement context based intervention to minimize barriers and missed opportunities on parent-adolescent communication on SRH issues. The government needs to build capacity in communities that emphasize the benefits of parent-adolescent communication on SRH issues. Individuals, communities and community leaders should promote and collaborate closely with local health staff in outreach activities in the communities.

Lastly, understanding the determinants of parent-adolescent communication on SRH issues is vital for parent-adolescent communication on SRH issues. And also identifies the area that needs to be focused by health care providers and policymakers. Furthermore, we need to explore new approaches that show promises, such as strengthening the private sector's engagement to reach adolescents and improve adolescents' access to reproductive health services and information.
